# Protein Tyrosine Phosphatases, TC-PTP, SHP1, and SHP2, Cooperate in Rapid Dephosphorylation of Stat3 in Keratinocytes Following UVB Irradiation

**DOI:** 10.1371/journal.pone.0010290

**Published:** 2010-04-22

**Authors:** Dae Joon Kim, Michel L. Tremblay, John DiGiovanni

**Affiliations:** 1 Department of Carcinogenesis, Science Park-Research Division, The University of Texas M. D. Anderson Cancer Center, Smithville, Texas, United States of America; 2 Department of Biochemistry, Rosalind and Morris Goodman Cancer Centre, McGill University, Montreal, Quebec, Canada; University of Illinois at Chicago, United States of America

## Abstract

Stat3 is initially dephosphorylated in murine keratinocytes in response to UVB irradiation. Treatment with Na_3_VO_4_ desensitized keratinocytes to UVB-induced apoptosis with the recovery of phosphorylated Stat3 protein levels, implying that a protein tyrosine phosphatase (PTP) is involved in this mechanism. In the current work, we report that three PTPs including TC45 (the nuclear form of TC-PTP), SHP1, and SHP2 are involved in this rapid dephosphorylation of Stat3 in keratinocytes induced by UVB irradiation. Dephosphorylation of Stat3 was increased rapidly after UVB irradiation of cultured keratinocytes. Knockdown of TC-PTP, SHP1, or SHP2 using RNAi showed that these PTPs are likely responsible for most of the rapid Stat3 dephosphorylation observed following UVB irradiation. The level of phosphorylated Stat3 was significantly higher in keratinocytes transfected with TC-PTP, SHP1, or SHP2 siRNA in the presence or absence of UVB compared with keratinocytes transfected with control siRNA. TC45 was mainly localized in the cytoplasm of keratinocytes and translocated from cytoplasm to nucleus upon UVB irradiation. Stat3 dephosphorylation was associated with nuclear translocation of TC45. Further studies revealed that knockdown of all three phosphatases, using RNAi, prevented the rapid dephosphorylation of Stat3 following UVB irradiation. In mouse epidermis, the level of phosphorylated Stat3 was initially decreased, followed by a significant increase at later time points after UVB exposure. The levels of Stat3 target genes, such as cyclin D1 and c-Myc, followed the changes in activated Stat3 in response to UVB irradiation. Collectively, these results suggest that three phosphatases, TC45, SHP1, and SHP2, are primarily responsible for UVB-mediated Stat3 dephosphorylation and may serve as part of an initial protective mechanism against UV skin carcinogenesis.

## Introduction

Signal transducer and activator of transcription 3 (Stat3) is one of a family of cytoplasmic proteins that participate as a transcription factor in the normal cellular responses to cytokines and growth factors [1], [2], [3], [4]. Upon activation by a wide variety of cell surface receptors, tyrosine phosphorylated Stat3 dimerizes and translocates to the nucleus and modulates the expression of target genes that are involved in various physiological functions including apoptosis (e.g., Survivin and Bcl-x_L_), cell cycle regulation (e.g., Cyclin D1, and c-Myc), and tumor angiogenesis (e.g., VEGF) [3], [5]. Studies have shown that constitutive activation of Stat3 is associated with a number of human tumors and cancer cell lines, including prostate, breast, lung, head and neck, brain, and pancreas, and its inhibition can suppress growth of cancer cells by promoting apoptosis and inhibiting cell proliferation [1], [2], [6], [7]. These data suggest that Stat3 plays a critical role in cancer cell proliferation and survival, making it a excellent target for therapeutic applications.

Recent studies of Stat3 have suggested that it has critical roles in skin carcinogenesis induced by chemicals and UVB [8], [9]. With regard to chemically-mediated skin carcinogenesis, Stat3-deficient mice were completely resistant to development of skin tumors induced by the two-stage carcinogenesis regimen and abrogation of Stat3 function by using a Stat3-specific decoy oligonucleotide inhibited the growth of skin tumors [10]. These and other studies [11], [12] have provided evidence that Stat3 is required for both the initiation and promotion stages of chemical carcinogenesis by maintaining survival of DNA-damaged stem/progenitor cells and by mediating cell proliferation necessary for the clonal expansion of initiated cells. Furthermore, using mice in which the expression of a constitutively active/dimerized form of Stat3 (Stat3C) is targeted to the proliferative compartment of epidermis via the bovine keratin 5 promoter (K5.Stat3C mice), we recently demonstrated heightened sensitivity to two-stage skin carcinogenesis compared to non-transgenic littermates [13]. In these mice, skin tumors that developed bypassed the premalignant stage and rapidly progressed to squamous cell carcinomas with characteristics of high vascularization, poor differentiation, and increased invasion. These results further confirmed a role for Stat3 in early stages of epithelial carcinogenesis and revealed a novel role of Stat3 in driving malignant progression of skin tumors in vivo [13].

In addition to its recently discovered roles in the development and progression of skin tumors induced by two-stage carcinogenesis, Stat3 also plays an important role in UVB-mediated skin carcinogenesis [8], [9]. In this regard, overexpression of Stat3 in keratinocytes reduced UVB-induced epidermal apoptosis, whereas Stat3-deficient keratinocytes were highly sensitive to UVB-induced apoptosis [14]. Interestingly, during the course of these studies we found that UVB irradiation initially caused rapid dephosphorylation of keratinocyte Stat3 and vanadate treatment desensitized keratinocytes to UVB-induced apoptosis with the recovery of phosphorylated Stat3 levels [14], suggesting the involvement of a tyrosine phosphatase. Furthermore, recent studies using mice either deficient in Stat3 or expressing constitutively active Stat3 in keratinocytes have confirmed a critical role for this transcription factor in UVB-mediated skin carcinogenesis [15].

In the current study, we show a critical role for three protein tyrosine phosphatases (PTPs); T-cell PTP (TC-PTP) and the Src homology 2 domain-containing PTPs, SHP1 and SHP2, in the dephosphorylation of Stat3 following UVB irradiation in skin. In addition, we show that all three PTPs are required for regulating the phosphorylation of Stat3 in skin keratinocytes in the absence of treatment with UVB. Finally, we show that nuclear translocation of TC-PTP occurs following UVB irradiation and contributes to rapid dephosphorylation of activated Stat3 by this PTP. Collectively, these results suggest that Stat3 dephosphorylation by TC-PTP, SHP1, and SHP2 in skin keratinocytes following exposure to UVB irradiation plays a role as an initial protective mechanism against UVB-induced skin carcinogenesis.

## Materials and Methods

### Ethics Statement

All experiments with animal were carried out with strict adherence to both institutional and National Institutes of Health (NIH) guidelines for minimizing distress in experimental animals. All experiments involving mice were approved by the University of Texas MD Anderson Cancer Center Institutional Animal Care and Use Committee (IACUC).

### Mice

Development of TC-PTP-deficient (TC-PTP-/-) and skin specific Stat3-deficient mice (K5Cre.Stat3^fl/fl^) has previously been described [16], [17]. Female transgenic and non-transgenic littermates at 7–8 weeks of age were used for the described experiments. The dorsal skin of each mouse was shaved 48 hours before UVB irradiation; only those mice in the resting phase of the hair cycle were used. For UVB irradiation, Westinghouse FS20 sun lamp bulbs with a peak emission at 313 nm were used. The fluence rate was measured with an IL1400A Radiometer/Photometer coupled to a SEL240/UVB-1/TD detector (International Light, Inc., Newburyport, MA). Each mouse was held in individual compartments of a plastic cage on a rotating base to prevent any differences in fluence across the UV light bulbs. An UVB-transparent lid covering the radiation chamber was used to filter out the small amount of UVC radiation emitted from these lamps.

### Keratinocyte cell culture

A previously described method [18] was used to culture keratinocytes obtained from 2 day-old neonates from wild-type FVB and TC-PTP-deficient mice. Primary keratinocytes, 3PC keratinocytes (an immortalized mouse keratinocyte cell line obtained from Ca^++^-resistant primary adult keratinocytes after exposure to 7,12-dimethylbenz[a]anthracene) [19] and HaCaT keratinocytes (Immortalized and nontumorigenic human skin keratinocyte cell line) [20] were cultured at 37°C and 5% CO_2_ in keratinocyte growth medium (Cambrex Inc., Walkersville, MD) containing 1% fetal bovine serum until 80 to 85% confluent at which time cells were irradiated with UVB at 800 J/m^2^. Prior to UVB irradiation, cultured keratinocytes were washed with DPBS two times to remove all remaining medium. A small volume of DPBS was added to cover keratinocytes under a thin layer of DPBS. Under this condition, cells were irradiated with UVB. For control keratinocytes, keratinocytes were treated identically, except for UVB irradiation. After UVB irradiation, DPBS was immediately removed and prewarmed medium was added to keratinocytes for additional culture before harvest.

### Protein isolation

Skin epidermis, primary keratinocytes, and 3PC keratinocytes (control and UVB treated) were used to prepare total cell lysates. For epidermal protein, mouse skin samples were placed on an ice-cold glass plate, and the epidermis was removed with a razor blade and placed into RIPA buffer containing 1% Triton X-100, protease inhibitor cocktail (Sigma-Aldrich Co., Saint Louis, MO), and phosphatase inhibitor cocktail I, II (Sigma-Aldrich Co.). For keratinocyte protein, cultured keratinocytes were washed with DPBS three times and placed into RIPA buffer by scraping. The lysates were incubated on ice for 20 min and then centrifuged at 14,000 g for 20 min at 4°C. The supernatant obtained from the high speed spin was used for the cytosolic fraction. Cytoplasmic and nuclear fractions were prepared from keratinocytes using NE-PER nuclear and cytoplasmic extraction reagents (Pierce Biotechnology Inc., Rockford, IL).

### Western blot analysis

Twenty-five to fifty µg of total protein from mouse epidermis or keratinocytes (control and UVB treated) were resolved using SDS-PAGE. For cytoplasmic and nuclear fractions, 30 µg of cytoplasmic protein or 10 µg of nuclear protein were resolved using SDS-PAGE. The samples were transferred onto a nitrocellulose membrane using an electroblotting method. The membrane was incubated overnight at 4°C with primary antibody, followed by incubation with a horseradish peroxidase-conjugated secondary antibody. Chemiluminescent detection reagents (GE Healthcare Bio-Sciences Co., Piscataway, NJ) were used to detect immunoreactive protein. The following primary antibodies were used: anti-phospho-Stat3 (Tyr705), anti-Stat3, anti-phospho-Stat1 (Tyr701), anti-Stat1, anti-cyclin D1, anti-Bcl-x_L_, anti-SHP1, anti-SHP2 (Cell Signaling Technology Inc., Beverly, MA), anti-phospho-Stat5A/B (Tyr694/699; Upstate, Lake Placid, NY), anti-Stat5 (BD Biosciences, San Jose, CA), anti-TCPTP 3E2 [21], anti-TC-PTP (R&D Systems Inc., Minneapolis, MN), anti-c-Myc and anti-Lamin A/C (Santa Cruz Biotechnology, Santa Cruz, CA), anti-lactate dehydrogenase (Chemicon International Inc., Temecula, CA), anti-actin (Sigma-Aldrich Co., Saint Louis, MO).

### Immunofluorescence analysis

Primary keratinocytes were grown on plastic chamber slides and were irradiated with UVB at 800 J/m^2^. One or two hours after UVB irradiation, keratinocytes were fixed with 100% methanol at −20°C for 15 min. After permeabilization with 0.5% Triton X-100 and blocking, cells were incubated with a monoclonal anti-TC-PTP antibody (R&D Systems Inc.) at 4°C for 12 h. Following extensive washing with PBS, cells were then incubated with Cy3-conjugated anti-mouse secondary antibody (Jackson ImmunoResearch Laboratories Inc., West Grove, PA) at 37°C for 1 h and mounted onto glass slides in Vectashield mounting medium with DAPI. For transfection, TC45-WT and TC45-D182A/Q262N substrate-trapping mutant were generated as described before [22], [23]. 3PC and HaCaT keratinocytes were grown on plastic chamber slides and were transfected with pcDNA4/myc-His-TC45-WT or pcDNA4/myc-His-TC45-D182A/Q262N substrate-trapping mutant using FuGENE 6 transfection reagent (Roche Applied Science, Indianapolis, IN). After 24 h of transfection, cells were irradiated with UVB at 800 J/m^2^. Two hours after UVB irradiation, keratinocytes were fixed with 100% methanol at -20°C for 15 min. Exogenous TC45 expression was detected by anti-myc antibody (Invitrogen, Carlsbad, CA).

### RNA interference (RNAi)

3PC keratinocytes were grown overnight to ∼40% confluence and transfected with ON-TARGETplus SMART pool short interfering RNA (siRNA) specific for mouse TC-PTP, , PTPRT, SHP1, SHP2, or ON-TARGETplus siCONTROL Non-targeting pool siRNA as a control (Dharmacon Inc., Layfayette, CO). Transfection was performed with Lipofectamine RNAiMAX (Invitrogen). Forty eight hours of siRNA transfection, cells were irradiated with UVB at 800 J/m^2^.

### PTPRT expression analysis

Total RNA was isolated from 3PC keratinocytes (control and UVB treated) according to the manufacturer's instructions and then further purified by Rneasy columns (Qiagen, Valencia, CA). RNA was resuspended in diethyl pyrocarbonate-treated water and quantified by spectrophotometry. One µg of RNA was reverse transcribed to cDNA by random priming. PTPRT expression was examined by PCR with primers 5′-AGTTCCAGCCTGCCATCCG-3′ and 5′-TCCAAAAGTCCTTCACCGTCT-3′ which are located in exons 16 and 20. The expected length of PCR product was 327 bp. β-casein was used as a control.

## Results

### PTP activity is critical for the regulation of Stat3 phosphorylation in keratinocytes

**Figure 1:**
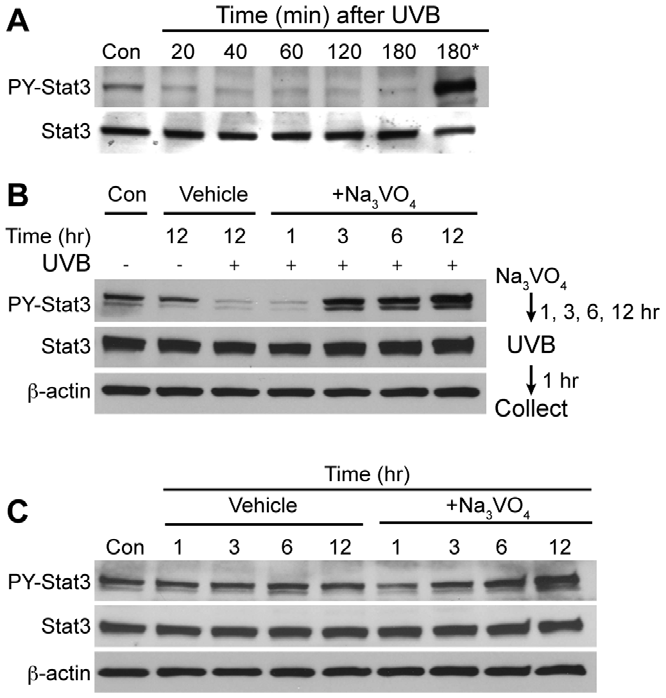
PTP inhibition leads to enhanced activation of Stat3 in keratinocytes. (A) Primary keratinocytes were cultured and irradiated with UVB at a dose of 800 J/m^2^. Cells were collected at the indicated time. For inhibition of PTP (lane 7, 180*), 100 µM of Na_3_VO_4_ was treated for 12 h before UVB irradiation. Cells were collected 3 h after UVB irradiation. Total cell lysates were resolved by SDS-PAGE and immunoblotted with antibodies specific for Stat3 and phosphorylated Stat3 (PY-Stat3). (B) 3PC keratinocytes were cultured with Na_3_VO_4_ for the indicated times (1, 3, 6, and 12 h) and then cells were exposed to UVB at a dose of 800 J/m^2^. One hour following UVB irradiation, cells were collected and processed for western blot analysis. Cells treated with vehicle only were harvested 1 h after UVB exposure (i.e. 13 h after vehicle treatment). (C) 3PC keratinocytes were cultured either in the presence or absence of Na_3_VO_4_ for the indicated time without UVB irradiation. Total cell lysates were resolved by SDS-PAGE and immunoblotted with antibodies specific for Stat3 and PY-Stat3. Con: control keratinocytes without UVB or vehicle.

As noted in the Introduction, previous studies indicated that Stat3 was dephosphorylated in keratinocytes after UVB irradiation [14]. In these studies, vanadate treatment desensitized keratinocytes to UVB-induced apoptosis with the recovery of phosphorylated Stat3 protein levels, implying involvement of a tyrosine phosphatase. In the current work, the mechanism whereby UVB exposure leads to rapid dephosphorylation of Stat3 was examined. Initially, keratinocytes were exposed to UVB irradiation and the level of Stat3 phosphorylation (tyr705) was determined. In this experiment, Stat3 was rapidly dephosphorylated even within 20 min of UVB exposure. Pretreatment with Na_3_VO_4_ for 12 h before UVB exposure significantly increased Stat3 phosphorylation at 3 h (180 min) compared to the level found in untreated keratinocytes (Fig. 1A). To further confirm increased phosphorylation of Stat3 in the absence of PTP activity, two additional experiments using Na_3_VO_4_ were performed. In the experiment shown in Figure 1B, Na_3_VO_4_ was given for various time periods prior to UVB exposure and then cells were harvested 1 h after UVB exposure. As shown, the level of phosphorylated Stat3 in keratinocytes was rapidly reduced in keratinocytes exposed to Na_3_VO_4_ for 1 h prior to UVB exposure (Fig. 1B). In contrast, cells exposed to Na_3_VO_4_ for 3 h or longer showed a protection against UVB-induced Stat3 dephosphorylation. Note that the level of phosphorylated Stat3 in keratinocytes after 3 h of Na_3_VO_4_ treatment was significantly higher compared to untreated keratinocytes (both control and vehicle treated cells, see Fig. 1B). These results further indicate that a phosphatase is activated very rapidly following UVB exposure and that vanadate must be available in the cell for more than 1 h in order to block UVB-mediated dephosphorylation of Stat3. As shown in Figure 1C, in the absence of UVB exposure, the level of phosphorylated Stat3 gradually increased with time in cells treated with Na_3_VO_4_ and by 6 and 12 h of Na_3_VO_4_ treatment was significantly higher compared to untreated keratinocytes (again see Fig. 1C). Collectively, the data in Figure 1 indicate that one or more PTP is critical for regulating Stat3 phosphorylation in the presence or absence of UVB irradiation.

### TC-PTP, SHP1, and SHP2 rapidly dephosphorylate Stat3 in response to UVB irradiation

**Figure 2:**
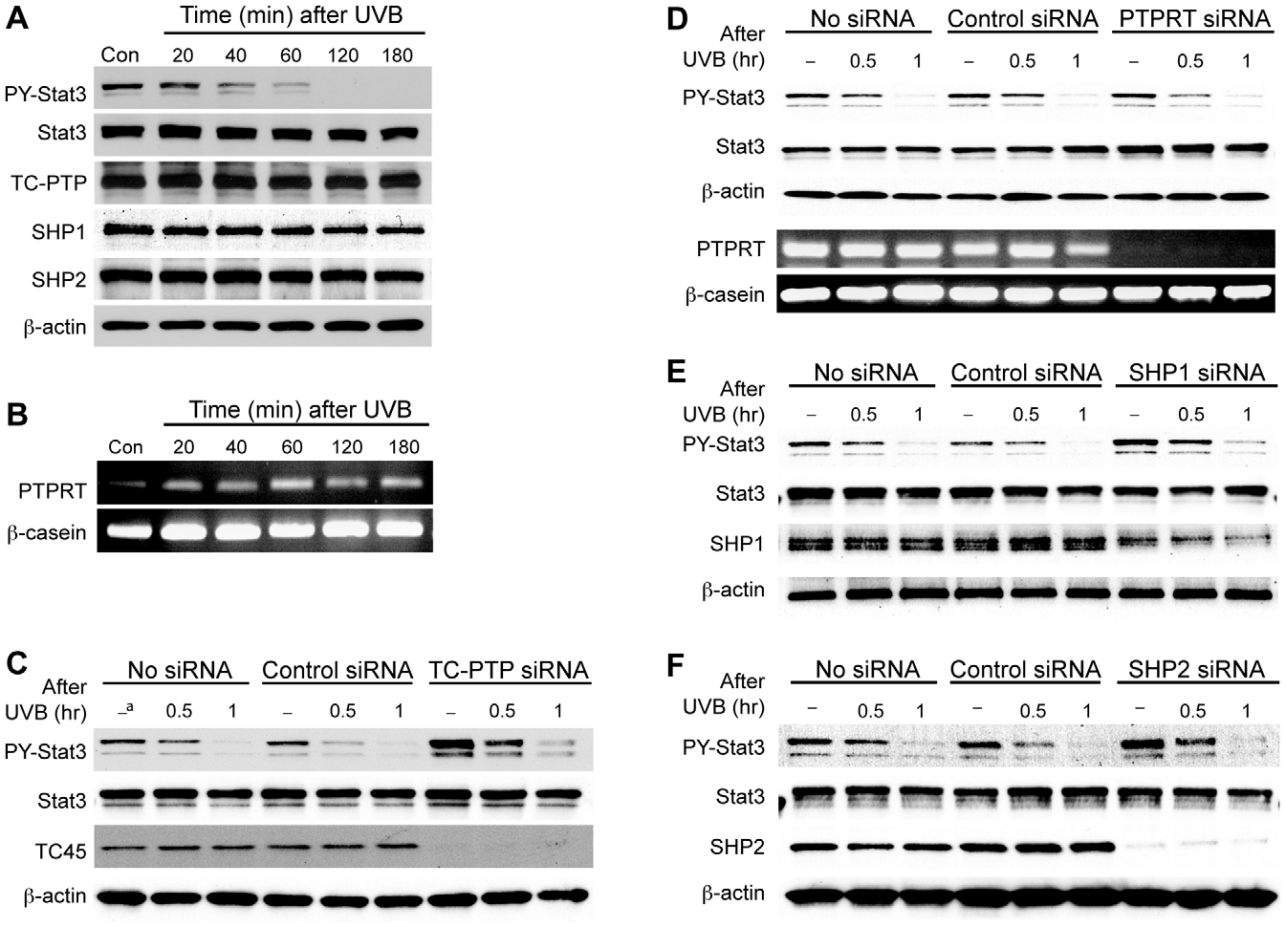
TC45, SHP1, and SHP2 rapidly dephosphorylates Stat3 after UVB irradiation *in vitro* mouse keratinocytes. (A) Western blot analysis of TC45, SHP1, and SHP2 in response to UVB irradiation. Mouse primary keratinocytes were cultured and irradiated with UVB at a dose of 800 J/m^2^. Cells were collected at the indicated time and total cell lysates were isolated. (B) Expression analysis of PTPRT by RT-PCR. Mouse primary keratinocytes were cultured and irradiated with UVB at a dose of 800 J/m^2^. Cells were collected at the indicated time and total RNA was isolated. (C-F) 3PC keratinocytes were transfected with siRNA specific for TC-PTP, PTPRT, SHP1, or SHP2. Cells were collected at the indicated time after UVB irradiation (800 J/m^2^). Total cell lysates were resolved by SDS-PAGE and immunoblotted with antibodies specific for Stat3, PY-Stat3. (C) Inhibition of TC-PTP expression by siRNA. (D) Inhibition of PTPRT expression by siRNA. (E) Inhibition of SHP1 expression by siRNA. (F) Inhibition of SHP2 expression by siRNA. ^a^ no UVB.

Previous studies have shown that Stat3 signaling is regulated by PTPs including LMW-DSP2 [24], TC-PTP [25], [26], [27], PTPRT [28], SHP1 [29], [30], SHP2 [31]. Among these PTPs, LMW-DSP2 negatively regulates IL6/LIF/Stat3-mediated signaling pathway in testicular cells. However, this PTP is not expressed in skin keratinocytes (data not shown). A nuclear form of TC-PTP, TC45, has been reported to play a role in the Jak/Stat signaling pathway by targeting Jak1, Jak3, Stat1, Stat3, and Stat5 [32], [33], [34]. Furthermore, recent studies suggested that TC45 has a potential role in the regulation of cell proliferation and cell cycle regulation [21], [25], [35], making TC45 a potential PTP candidate for UVB-mediated Stat3 dephosphorylation in skin keratinocytes. Western blot analysis showed that while the level of phosphorylated Stat3 was rapidly reduced in response to UVB irradiation, TC45 was constitutively expressed in murine keratinocytes independent of UVB irradiation (Fig. 2A). The cytoplasmic form of TC-PTP, TC48, was not detected in murine keratinocytes, which is consistent with a previous report [36], [37]. SHP1 mediates the inhibition of Stat3 signaling induced by either butein or boswellic acid in multiple myeloma cells [29], [30]. SHP2 regulates neurogenesis by regulating Erk and Stat3 signaling pathways [31]. Both cytoplasmic PTPs were also constitutively expressed in murine keratinocytes independent of UVB irradiation (See again Fig. 2A). PTPRT reportedly dephosphorylates Stat3 in colorectal cell lines, even though it is a receptor type PTP [28]. PTPRT was also expressed in murine keratinocytes independent of UVB irradiation (Fig. 2B).

To identify a PTP (or PTPs) involved in Stat3 dephosphorylation in skin keratinocytes in response to UVB irradiation, knockdown of each of the above mentioned PTPs was performed using RNAi. As shown in Fig. 2C, dephosphorylation of activated Stat3 was reduced in 3PC keratinocytes transfected with TC-PTP siRNA after UVB irradiation compared to control 3PC keratinocytes without transfection or 3PC keratinocytes transfected with control siRNA. However, Stat3 was still dephosphorylated in response to UVB irradiation even in the presence of significantly reduced levels of TC45 (Fig. 2C and S1A). In particular, the level of phosphorylated Stat3 in keratinocytes transfected with TC-PTP siRNA was higher compared to control 3PC keratinocytes transfected without transfection and 3PC keratinocytes transfected with control siRNA. The impact of TC-PTP knockdown on Stat3 phosphorylation was higher in keratinocytes in the absence of UVB irradiation compared with keratinocytes after UVB irradiation (Fig. 2C and S1A). Knockdown of PTPRT using siRNA showed that the level of phosphorylated Stat3 was similarly reduced in 3PC keratinocytes transfected with PTPRT siRNA after UVB irradiation compared to control 3PC keratinocytes without transfection and 3PC keratinocytes transfected with control siRNA (Fig. 2D). These data indicate that PTPRT is not involved in the rapid Stat3 dephosphorylation that occurs following UVB exposure. Similar to TC-PTP siRNA, knockdown of either SHP1 or SHP2 in keratinocytes reduced dephosphorylation of activated Stat3 in response to UVB irradiation compared to control 3PC keratinocytes. (Figures 2E and S1B, and Figure 2F and S1C, respectively). In particular, the level of phosphorylated Stat3 in keratinocytes transfected with either SHP1 or SHP2 siRNA was higher compared to control 3PC keratinocytes transfected without transfection and 3PC keratinocytes transfected with control siRNA. Its effect on Stat3 phosphorylation was also higher in keratinocytes in the absence of UVB irradiation compared with keratinocytes after UVB irradiation (Figures 2E and S1B, and Figure 2F and S1C, respectively). These results suggest that at least three PTPs (i.e., TC45, SHP1 and SHP2) are involved in dephosphorylation of Stat3 in the presence and absence of UVB irradiation.

### TC45 primarily localizes in the cytoplasm and translocates to the nucleus in keratinocytes after UVB irradiation

**Figure 3:**
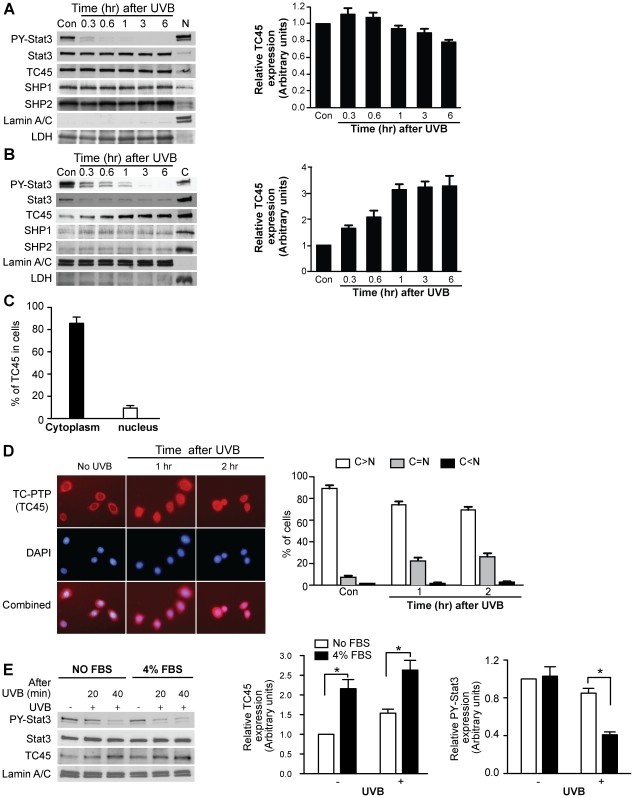
TC45 primarily localizes to cytoplasm and translocates to the nucleus after UVB irradiation in keratinocytes. (A and B) Primary keratinocytes were cultured and irradiated with UVB at a dose of 800 J/m^2^. Cells were collected at the indicated time. Cytoplasmic and nuclear lysates were separately isolated. (A) Western blot analysis of cytoplasmic phosphorylated Stat3, TC45, SHP1, and SHP2 after UVB exposure in keratinocytes. N: nuclear fraction, 0 h. LDH was used as a control for cytoplasmic fraction. Right: Relative expression levels of TC45 were quantified by densitometry. Results are the mean + standard deviation from three independent experiments. (B) Western blot analysis of nuclear phosphorylated Stat3, TC45, SHP1, and SHP2 after UVB exposure in keratinocytes. C: cytoplasmic fraction, 0 h. Lamin A/C was used as a control for nuclear fraction. Right: Relative expression levels of TC45 were quantified by densitometry. Results are the mean + standard deviation from three independent experiments. (C) Quantification of nuclear and cytoplasmic TC45 in keratinocytes without UVB irradiation. Values represent the percentage of the quantified signals (Land 1 and 7) shown in (B) relative to loading volume and total protein yield obtained from both nuclear and cytoplasmic fractions. Results are the mean + standard deviation from three independent experiments. (D) Immunofluorescence analysis of nuclear translocation of TC45 after UVB irradiation. Primary keratinocytes were cultured and irradiated with UVB at a dose of 800 J/m^2^. Keratinocytes were subjected to immunofluorescence analysis by using an anti-TC-PTP antibody. The nuclei were visualized by DNA staining with DAPI (blue). Right: Quantification of TC45 localization. Subcellular localization of TC45 was scored according to whether it was higher in the cytoplasm (C > N), evenly distributed between cytoplasm and nucleus (C  =  N), or higher in the nucleus (C < N) for 200 cells at each time point. Results are the mean + standard deviation from three independent experiments. (E) Western blot analysis of nuclear phosphorylated Stat3 and TC45 after UVB exposure in keratinocytes. 3PC keratinocytes were cultured in serum free keratinocyte growth medium or medium containing 4% FBS, and irradiated with UVB at a dose of 800 J/m^2^. Relative expression levels of TC45 and phosphorylated Stat3 after 20 min of UVB exposure were quantified by densitometry. Results are the mean + standard deviation from three independent experiments.

Stat3 localizes in the cytoplasm as an inactive form. After activation, tyrosine phosphorylated Stat3 translocates to the nucleus. Therefore, the cellular localization of TC45, SHP1 and SHP2 was investigated in keratinocytes following UVB irradiation. As expected, both SHP1 and SHP2 were predominantly localized in the cytoplasm compared to nucleus (Fig. 3A and B). Their cellular distribution was not changed following UVB irradiation (Fig. 3A and B). TC45 contains a bipartite nuclear localization signal and is reported to be primarily localized in the nucleus [38], [39]. Previous studies showed that TC45 accumulates in the cytoplasm in response to cellular stresses, such as hyperosmotic stress, and dephosphorylates the epidermal growth factor receptor in the cytoplasm [40]. Interestingly, in mouse keratinocytes, TC45 was mainly localized to cytoplasm (Fig. 3A) and then was translocated from cytoplasm to nucleus in response to UVB irradiation as shown in western blot analysis (Fig. 3B). In the absence of UVB treatment, almost 90% of TC45 was localized to the cytoplasm, whereas only 10% of TC45 was in the nucleus (Fig. 3C). TC45 was also primarily localized in the cytoplasm of mouse epidermis (data not shown). Dephosphorylation of Stat3 in the nucleus was rapidly increased after UVB irradiation of cultured keratinocytes and associated with nuclear accumulation of TC45 (see again Fig. 3B). The data in Figures 3A and 3B demonstrate that activated Stat3 is dephosphorylated initially in the cytoplasm in response to UVB irradiation prior to translocation to the nucleus. However, rapid dephosphorylation of nuclear Stat3 also occurred and was associated with accumulation of TC45. Immunofluorescence analysis confirmed that TC45 localized primarily to cytoplasm in untreated cells, especially in the perinuclear region, and then rapidly translocated to the nucleus following UVB exposure (Fig. 3D).

It is possible that growth factors (e.g. TGFα) might increase nuclear translocation of TC45 because Stat3 is activated by growth factor signaling in keratinocytes [8], [41]. In addition, UVB exposure of keratinocytes is known to upregulate ligands for the EGFR and lead to its rapid activation [42], [43]. To investigate the effect of growth factors on nuclear translocation of TC45, 3PC keratinocytes were cultured in serum free medium or medium containing 4% serum and then irradiated with UVB. Both the level of nuclear TC45 and its nuclear translocation following UVB irradiation were increased in cells cultured with serum-containing medium (Fig. 3E). Furthermore, Stat3 dephosphorylation was accelerated with increased nuclear translocation of TC45 following UVB irradiation (see again Fig. 3E). Collectively, these results suggest that nuclear translocation of TC45 contributes to the rapid dephosphorylation of Stat3 in mouse keratinocytes following UVB exposure and that enhanced growth factor signaling further enhances this process.

**Figure 4:**
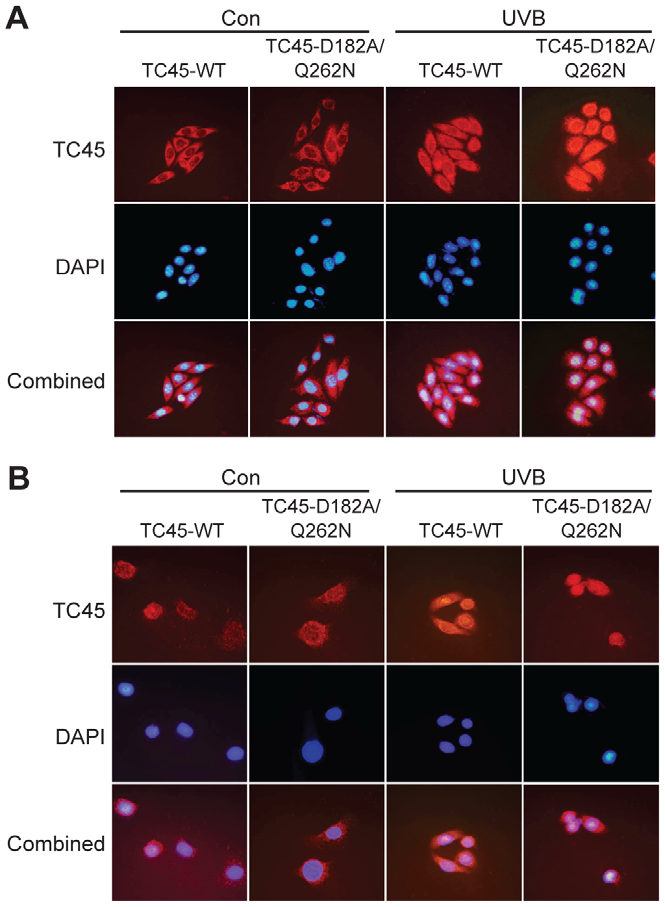
Nuclear translocation of TC45 in response to UVB irradiation is independent of phosphatase activity in keratinocytes. (A) 3PC and (B) HaCaT keratinocytes were transfected with plamid vectors expressing TC45-WT or TC45-D182A/Q262N. After 2 h of UVB irradiation (800 J/m^2^), keratinocytes were subjected to immunofluorescence analysis by using an anti-myc antibody to detect exogenous TC45 expression. The nuclei were visualized by DNA staining with DAPI (blue). Right: Quantification of TC45 localization. Results are the mean + standard deviation from three independent experiments.

Transfection analysis using both TC45-WT and the substrate-trapping mutant TC45-D182A/Q262N, lacking phosphatase activity by substituting its essential catalytic residues, confirmed its nuclear translocation following UVB exposure. In 3PC keratinocytes expressing TC45-WT, TC45 is primarily localized to cytoplasm and translocated to the nucleus following UVB exposure (Fig. 4A). The TC45-D182A/Q262N mutant is also similarly translocated to the nucleus, indicating nuclear translocation is independent of its phosphatase activity (Fig. 4A). In transfected human HaCaT keratinocytes, both TC45-WT and TC45-D182A/Q262N mutant were also localized to cytoplasm and translocated to the nucleus following UVB exposure. However, TC45 was expressed to a greater extent in the nucleus of human keratinocytes compared to mouse keratinocytes under the same culture conditions (Fig. 4B).

### TC45 deficiency leads to reduced Stat3 dephosphorylation in response to UVB irradiation

**Figure 5:**
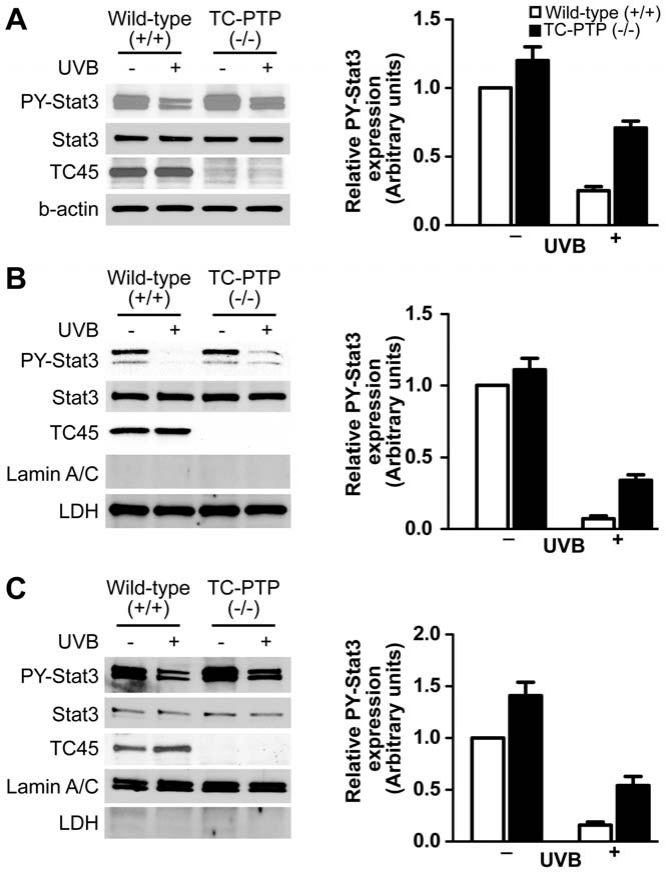
TC-PTP deficiency retards Stat3 dephosphorylation after UVB exposure. (A) Western blot analysis of phosphorylated Stat3 in total cell lysates of TC-PTP-deficient primary keratinocytes. Wild-type and TC-PTP-deficient primary keratinocytes were cultured and irradiated with UVB at a dose of 800 J/m^2^. Cells were collected after 1 h of UVB irradiation. Western blot analysis of (B) cytoplasmic and (C) nuclear phosphorylated Stat3 in TC-PTP-deficient primary keratinocytes. Wild-type and TC-PTP-deficient primary keratinocytes were cultured and irradiated with UVB at a dose of 800 J/m^2^. Cells were collected after 1 h of UVB irradiation. Cytoplasmic and nuclear lysates were separately isolated. Relative levels of phosphorylated Stat3 were quantified by densitometry. Results are the mean ± standard deviation from three independent experiments.

Studies using keratinocytes from TC-PTP-deficient mice [16] also showed results similar to those observed with TC-PTP siRNA. The level of phosphorylated Stat3 in TC-PTP-deficient keratinocytes was significantly higher compared with the level seen in wild-type keratinocytes without UVB irradiation and its reduction after UVB irradiation was partially reversed compared to wild-type keratinocytes (Fig. 5A). These results provide further evidence that TC45 is a critical PTP involved in regulating Stat3 phosphorylation in keratinocytes. Further studies showed that both nuclear and cytoplasmic TC45 are involved in rapid dephosphorylation of Stat3. In this regard, similar to whole cell lysates, the level of phosphorylated Stat3 in TC-PTP-deficient keratinocytes was also higher in both cytoplasm and nucleus compared to wild-type keratinocytes without UVB irradiation (Fig. 5B and C). However, the effect of TC45 deficiency on Stat3 dephosphorylation was significantly higher in nucleus compared to cytoplasm, even though the majority of TC45 is localized in cytoplasm as shown in Figure 3. These results further indicate that nuclear translocation of TC45 is an important mechanism for Stat3 dephosphorylation in both untreated as well as UVB treated keratinocytes.

### TC45, SHP1, and SHP2 cooperate in dephosphorylation of Stat3 in response to UVB irradiation

**Figure 6:**
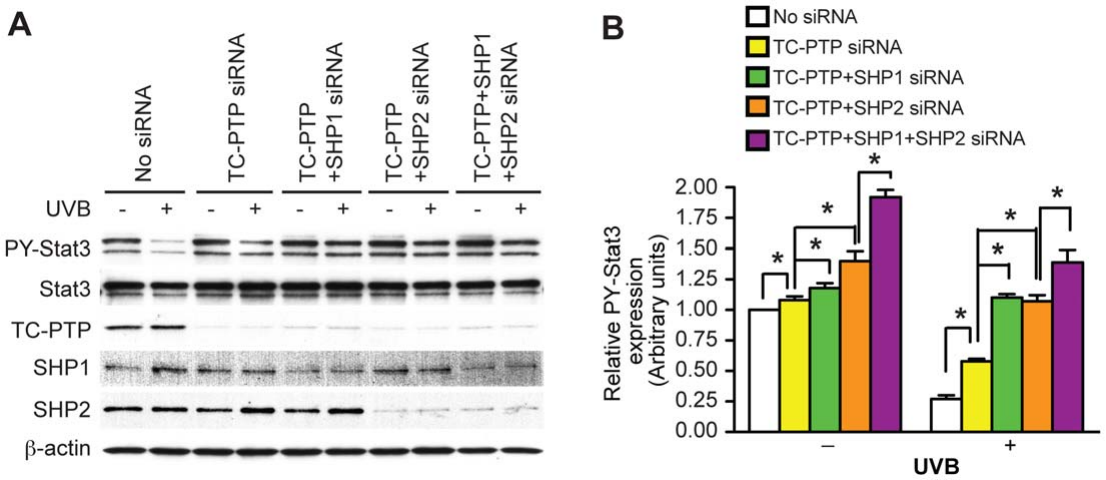
TC45, SHP1, and SHP2 cooperatively recover the level of phosphorylated Stat3 in response to UVB irradiation. 3PC keratinocytes were transfected with siRNA specific for TC-PTP, SHP1, and SHP2. Cells were collected after 1 h of UVB irradiation (800 J/m^2^). Total cell lysates were resolved by SDS-PAGE and immunoblotted with antibodies specific for Stat3, PY-Stat3. Lane 1 and 2, No siRNA; Land 3 and 4, Inhibition of TC-PTP expression by siRNA; Lane 5 and 6, Inhibition of TC-PTP and SHP1 expressions by siRNAs; Lane 7 and 8, Inhibition of TC-PTP and SHP2 expressions by siRNAs; Lane 9 and 10, Inhibition of TC-PTP, SHP1, and SHP2 expressions by siRNA. Relative levels of phosphorylated Stat3 were quantified by densitometry. Results are the mean + standard deviation from three independent experiments.

TC45, SHP1 and SHP2, are involved in UVB-mediated Stat3 dephosphorylation, respectively (See again Fig. 2). To examine whether these PTPs have synergistic effects on Stat3 dephosphorylation after UVB irradiation, two PTPs (TC-PTP and SHP1, TC-PTP and SHP2) or all three PTPs were simultaneously knocked down in mouse keratinocytes using RNAi. As shown in Figure 6, simultaneous knockdown of either TC-PTP and SHP1, or TC-PTP and SHP2 in keratinocytes reduced Stat3 dephosphorylation to a greater extent following UVB irradiation compared to keratinocytes that only had knockdown of TC-PTP. Furthermore, simultaneous knockdown of all three PTPs in keratinocytes reduced dephosphorylation of Stat3 in response to UVB irradiation to a greater extent compared to knockdown of only two PTPs as well as knockdown of TC-PTP only (Fig. 6). In this regard, the level of phosphorylated Stat3 was significantly increased in keratincytes transfected with siRNAs of two PTPs compared with the level seen in keratinocytes transfected with TC-PTP siRNA only (Fig. 6). This was further enhanced with siRNAs of all three PTPs (See again Fig. 6). These results suggest that activation of three PTPs cooperated in dephosphorylation of Stat3 in mouse keratinocytes following UVB irradiation.

### Stat3 dephosphorylation is an initial protective mechanism that occurs rapidly after UVB irradiation

**Figure 7:**
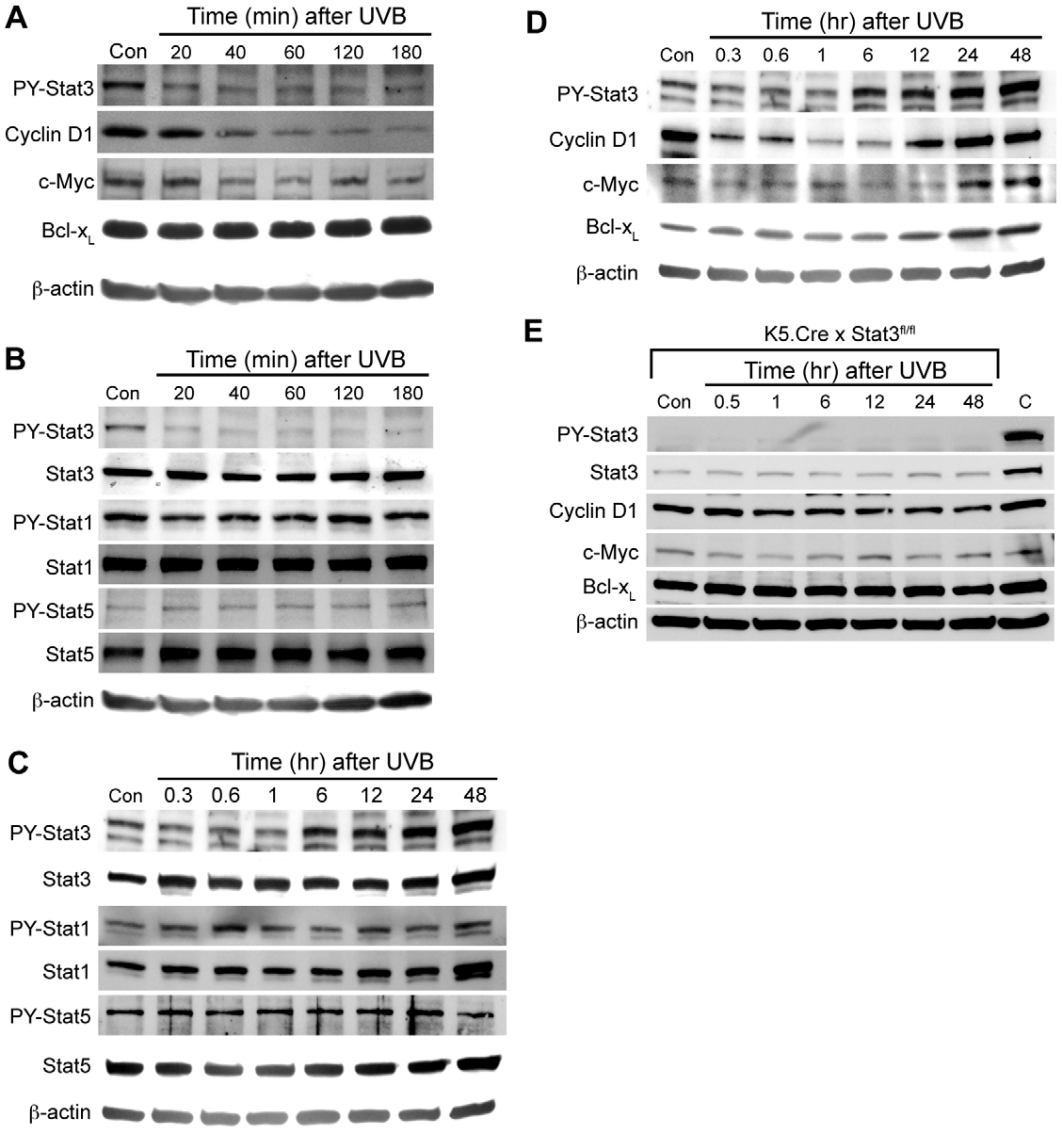
UVB-induced Stat3 dephosphorylation is an initial protective mechanism occurred against UVB exposure. (A-B) Mouse primary keratinocytes were cultured and irradiated with UVB at a dose of 800 J/m^2^. Cells were collected at the indicated time and total cell lysates were isolated. (A) Western blot analysis of Stat3-mediated target proteins in response to UVB irradiation. (B) Western blot analysis of phosphorylated Stat1, Stat3 and Stat5 in response to UVB irradiation. (C-E) Mice (n = 3) were irradiated with UVB at a dose of 1,000 J/m^2^ and total epidermal extracts were isolated at the indicated time point after UVB irradiation. (C) Western blot analysis of phosphorylated Stat1, Stat3, and Stat5 in response to UVB irradiation. (D) Western blot analysis of Stat3-mediated target proteins in response to UVB irradiation. (E) Western blot analysis of Stat3-mediated target proteins in the epidermis of Stat3-deficient mice. Stat3-deficient mice (n = 3) were irradiated with UVB at a dose of 1,000 J/m^2^ and total epidermal extracts were isolated at the indicated time point after UVB irradiation. C: wild-type mice, untreated.

Activated Stat3 modulates the expression of target genes that are involved in cell proliferation and survival. Western blot analysis of Stat3 target proteins showed cyclin D1 and c-Myc were downregulated following UVB-induced dephosphorylation of Stat3 in keratinocytes (Fig. 7A). However, the level of Bcl-x_L_, an antiapoptotic gene that is a Stat3 target, was not changed (Fig. 7A). This indicates that other signaling mechanisms involved in the regulation of Bcl-x_L_ expression compensate for the loss of Stat3-mediated signaling that occurs after UVB irradiation [44]. TC45 is also known to dephosphorylate other Stat family members such as Stat1 and Stat5. However, as shown in Fig. 7B, Stat1 and Stat5 were not dephosphorylated in response to UVB irradiation in cultured keratinocytes.

Following irradiation of mouse skin with UVB, the level of phosphorylated Stat3 was initially decreased and then increased significantly at later time points in epidermis (Fig. 7C). Similar to the pattern of phosphorylated Stat3, the levels of cyclin D1 and c-Myc protein were initially decreased and then increased (Fig. 7D). Again, Bcl-x_L_ protein level was not initially downregulated, but increased at later time points after UVB exposure, similar to cyclin D1 and c-Myc (Fig. 7D). These later increases coincided with activation of Stat3. To further examine whether these changes in target protein levels were dependent, at least in part, on Stat3-mediated signaling in response to UVB irradiation, skin specific Stat3-deficient mice (K5Cre.Stat3^fl/fl^) were irradiated with UVB. The levels of cyclin D1, c-Myc, Bcl-x_L_ proteins were decreased in the epidermis of untreated K5Cre.Stat3^fl/fl^ mice compared to control wild-type mice as previously shown (Fig. 7E) [10], [11]. Following UVB treatment, additional decreases in protein levels of cyclin D1 and c-Myc were observed in K5Cre.Stat3^fl/fl^ mice after UVB irradiation (Fig. 7E), although these decreases did not appear as great as in wild-type mice. Furthermore, the protein levels of all three target genes (including Bcl-x_L_) were not increased at later time points following UVB exposure (Fig. 7E), indicating that the later UVB-induced changes in levels of cyclin D1, c-Myc, and Bcl-x_L_ are also dependent in part on Stat3 signaling. Similar to cultured keratinocytes, Stat1 and Stat5 were not dephosphorylated in response to UVB irradiation in mouse epidermis (Fig. 7C). Collectively, these results suggest that the rapid dephosphorylation of Stat3 following exposure of keratinocytes to UVB is mediated by three distinct PTPs, TC45, SHP1 and SHP2, and that changes in target gene expression are dependent at least in part (except Bcl-x_L_ early after UVB) on the phosphorylation changes observed in Stat3.

## Discussion

Tyrosine phosphorylation of proteins catalyzed by protein tyrosine kinases (PTKs) plays a critical role in regulating proliferation, differentiation, and survival, and abnormalities in protein phosphorylation are associated with various human diseases including cancer. Tyrosine phosphorylation by PTKs is balanced by PTPs that regulate the rate and duration of the signaling responses that are amplified by PTKs [36], [45], [46]. Growing evidence indicates that PTPs have specific and active roles in the regulation of signaling mechanisms, rather than being simple housekeeping enzymes [47], [48]. The fine tuning of cell signaling by balanced actions of PTKs and PTPs is crucial in maintaining cellular homeostasis and protecting cells from exogenous factors, like chemicals or UV radiation.

In the current study, we show that Stat3 is rapidly dephosphorylated in both nucleus and cytoplasm of keratinocytes upon UVB exposure. TC-PTP, SHP1, and SHP2 are the primary PTPs that contribute to rapid Stat3 dephosphorylation in response to UVB irradiation in skin keratinocytes. We show for the first time that TC45 is mainly localized to cytoplasm of both mouse and human keratinocytes. In addition, nuclear translocation of TC45 contributed to UVB-mediated dephosphorylation of Stat3 in mouse keratinocytes. Finally, all three PTPs also appear to play a role in normal regulation of Stat3 phosphorylation in unexposed keratinocytes. In our previous studies, we showed that the level and/or activation state of Stat3 dramatically influenced the response of keratinocytes to UVB. In this regard, Stat3 deficiency enhanced UVB-induced apoptosis whereas Stat3 activation (i.e., overexpression of a constitutively active form of Stat3, Stat3C) reduced UVB-induced apoptosis in mouse keratinocytes in culture or mouse keratinocytes in vivo [14], [15]. The rapid dephosphorylation of Stat3 that occurs following UVB would serve to increase susceptibility to UVB-mediated apoptosis and thereby limit survival of keratinocytes with DNA damage that could lead to mutations and cancer development. It is interesting to note that after the initial dephosphorylation of Stat3 in vivo, phospho-Stat3 levels rise (see Figure 7C and reference 14). We have shown that Stat3 activation is required for skin tumor formation following UVB exposure [15]. Thus, although rapid dephosphorylation of Stat3 by three PTPs may initially serve to protect skin from UVB-mediated carcinogenesis, this mechanism is overcome by other effects induced by UVB that ultimately lead to activation of Stat3 and tumor development [8], [9], [14], [15].

As shown in this study, TC45, SHP1, and SHP2 contributed to dephosphorylation of Stat3 in the absence or presence of UVB exposure. However, the results shown in Figure 6 indicated that Stat3 was still dephosphorylated to some extent following exposure to UVB in cells deficient in all three PTPs. There are two possible explanations for these results. First, siRNA knockdown may not be sufficient to completely inhibit activation of one or more of the three PTPs after UVB irradiation. It is possible that UVB-mediated activation of PTPs that still remained after siRNA knockdown could contribute to residual Stat3 dephosphorylation. Second, one or more unidentified PTP(s) activated by UVB irradiation contributed to the rapid dephosphorylation of Stat3 in addition to TC45, SHP1 and SHP2. Further work is necessary to completely explore these possibilities. In addition, the results shown in Figure 6 indicate that the effect of siRNA knockdown of all three PTPs was much greater compared with siRNA knockdown of each PTP on Stat3 phosphorylation after UVB irradiation. It is possible that the absence or reduction of each PTP can be compensated by activation of other two PTPs after UVB irradiation.

Subcellular localization of PTPs is one of the critical factors in determining substrate specificity and subsequent signaling specificity, in addition to side-chain interactions between the substrates and areas flanking the PTP active site [49], [50]. To date, TC45 has been reported to be primarily localized in the nucleus of cells. Furthermore, most of this work has been done in cell lines such as COS1, HeLa, and NIH3T3 cells [33], [40], [51]. In the current studies, we show that TC45 is predominantly localized in cytoplasm of skin keratinocytes and translocates to the nucleus following UVB exposure (Fig. 3 and 4). Thus, TC45-mediated signaling regulation in keratinocytes may be somewhat different from other cells and tissues where TC45 is mainly localized in nucleus. In addition, UVB-induced nuclear translocation of TC45 suggests that skin-specific factor(s) expressed in cytoplasm may bind to TC45 in keratinocytes and prevent its nuclear location and activity. UVB irradiation may cause separation of these factors from TC45, and then make it possible for nuclear translocation to occur leading to dephosphorylation of Stat3. Further studies related to this hypothesis are currently in progress.

Stat3 has critical roles in cancer cell proliferation and survival and its constitutive activation is associated with a number of human tumors. However, the fact that naturally occurring mutations of Stat3 leading to its constitutive activation have not been identified implies aberrant growth factor signaling may play an important role in the constitutive activation of Stat3, rather than alterations in the gene per se [2]. Recent genome-wide studies showed that single nucleotide polymorphisms on chromosome 18p11 encoding TC-PTP are associated with the development of several chronic inflammatory diseases including Crohn's disease [52], [53], although genetic mutations have not been identified in human cancers to date. Collectively, our studies suggest that inactivation of phosphatases such as TC-PTP due to aberrant signaling pathways could lead to constitutive activation of Stat3, contributing to the development of cancers including skin cancer.

## Supporting Information

Figure S1 Quantification of phosphorylated Stat3 in keratinocytes following siRNA-mediated inhibition of PTP expression. Relative levels of phosphorylated Stat3 were quantified by densitometry. Results are the mean + standard deviation from three independent experiments. (A) Inhibition of TC-PTP expression by siRNA. (B) Inhibition of SHP1 expression by siRNA. (C) Inhibition of SHP2 expression by siRNA. Differences between no siRNA (or control siRNA) and each PTP siRNA were significant (p<0.05) by student t test.
(0.85 MB TIF)
