# Supplementary figures and images for: Protein Tyrosine Phosphatases, TC-PTP, SHP1, and SHP2, Cooperate in Rapid Dephosphorylation of Stat3 in Keratinocytes Following UVB Irradiation
Source: PLoS One. 2010 Apr 22;5(4):e10290. doi: 10.1371/journal.pone.0010290 (PMC2858656; doi:10.1371/journal.pone.0010290)

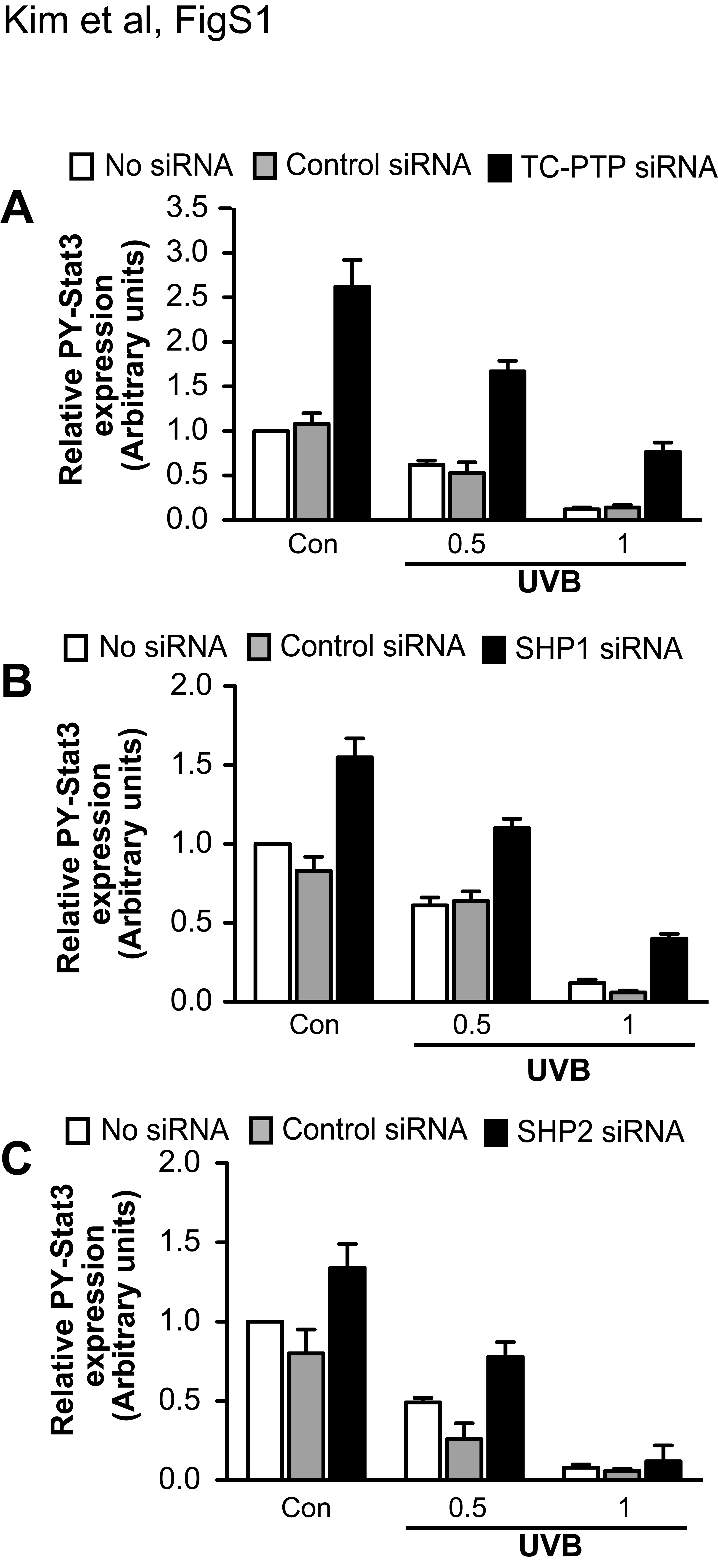

Supplement: Figure S1 — Quantification of phosphorylated Stat3 in keratinocytes following siRNA-mediated inhibition of PTP expression. Relative levels of phosphorylated Stat3 were quantified by densitometry. Results are the mean + standard deviation from three independent experiments. (A) Inhibition of TC-PTP expression by siRNA. (B) Inhibition of SHP1 expression by siRNA. (C) Inhibition of SHP2 expression by siRNA. Differences between no siRNA (or control siRNA) and each PTP siRNA were significant (p<0.05) by student t test. (0.85 MB TIF) [file pone.0010290.s001.tif]
